# Centers for medicare & medicaid services radiation oncology alternative payment model and the future of radiation oncology physics practice

**DOI:** 10.1002/acm2.12823

**Published:** 2020-01-28

**Authors:** Michael Mills, Tim Solberg, Per Halvorsen

**Affiliations:** ^1^ University of Louisville Louisville Kentucky USA; ^2^ University of California, San Francisco San Francisco CA USA; ^3^ Lahey Health Burlington MA USA

There are still a few of us that remember the state of medical physics practice in the early 1990s, and what a difficult time it was. There was no definition of Qualified Medical Physicist (QMP); so, almost anyone with a related degree could claim to be a medical physicist. There were a few with newly minted PhD degrees in a related field who took radiological physics and dosimetry short courses and immediately found jobs where someone would train them. The only staffing recommendations were found in the ACR “Blue Book,” which recommended one chief and one additional staff physics member per 400 new radiation oncology patients per year. There were no accredited medical physics residency programs of any kind. The Abt Reports on workforce and staffing did not exist, and there was no easy way for medical physicists to defend themselves from overwork. There was no JACMP, thus, no easy way for medical physicists to share clinical solutions. In many cases, we reinvented the wheel and solved the same clinical problem at many clinics around the nation. Good jobs were hard to find, and there was a reluctance to change jobs out of fear of moving from a bad position to worse.

In short, we had little identity, no accredited clinical training, no clinical journal, no way to defend ourselves from overwork, and few good job opportunities. Things are so much better now, right? For the moment, things are very gratifying. Thanks to the hard work of many dedicated scholars, we have the kind of profession we only dreamed of in 1990. However, on our blind side, there is a freight train bearing down on us, and we are tied to the railroad tracks. We could be headed to a situation just as bad or worse. Or, hopefully, perhaps not; let us engage in a thought exercise to explore the meaning of what is to come. Maybe together we can meet the challenge in a creative way.

In the 1990s, as well as today, clinical radiation oncology physicists have been and are reimbursed under the CPT 77300 series technical components. The challenge was to measure clinical physicist work and staffing based on these CPT code charges. In 1994, a study was designed to measure clinical physicist work in the same way that physician work was measured for the CPT code professional component. Preservice work (not patient specific), intraservice work, and postservice work (both patient specific) were captured along with the service mix and staffing for a wide range of providers. AAPM and ACMP provided expert physicist personnel and contracted with Abt Associates, Inc. to conduct the research and report on its findings. The four Abt reports provided a straightforward method for physicists to defend their workforce, revenue stream, and staffing levels for over two decades (1995–2020).

In 2012 and later updated in 2019, ASTRO, along with endorsement by a number of other organizations, published two reports: “Safety is no Accident: A Framework for Quality Radiation Oncology Care.” (https://www.astro.org/Patient-Care-and-Research/Patient-Safety/Safety-is-no-Accident); (https://www.astro.org/uploadedFiles/_MAIN_SITE/Daily_Practice/Accreditation/Content_Pieces/SafetyisnoAccident.pdf). These documents contain a detailed grid on physics staffing and have the support of a wide section of the radiation oncology community, including physicians. This is important because physicians have historically been important advocates for the physicist’s contributions to safe practice.

The workload associated with the Abt and *Safety is no Accident* (SINA) studies was based on time and effort by CPT code service mix, patient load categories, and measured staffing. However, now the clinical physics community is facing a significant challenge of far greater complexity than it faced in the early 1990s. A future Abt V study likely will not be able to meet this challenge.

In July 2019, the Centers for Medicare & Medicaid Services (CMS) proposed a “Radiation Oncology Alternative Payment Model” (RO‐APM) as a major step for transitioning from volume‐based to value‐based healthcare. In September 2019, the AAPM submitted comments to CMS addressing their appreciation and concerns with the APM. (https://www.regulations.gov/document?D=CMS-2019-0101-0158). The CMS document was entitled: “Medicare Program: Specialty Care Models to Improve Quality of Care and Reduce Expenditures; CMS‐5527‐P.” There is, therefore, an explicit goal to increase the value of CMS healthcare purchases by reducing expenses.(https://www.federalregister.gov/documents/2019/07/18/2019-14902/medicare-program-specialty-care-models-to-improve-quality-of-care-and-reduce-expenditures)

So, this is the train, and this is the landscape: Beginning in the late 1990s, the IMRT reimbursement bubble was responsible for driving physicist demand, and in turn, physicist training, credentialing, and compensation. Even so, today, in many venues, CPT codes underrepresent physics effort, and physics CPT revenue does do not cover salaries and benefits for the physics effort at current levels. (Likewise, CPT professional codes do not cover physician salaries.) Physicists must reinvent themselves, as they are currently doing, by focusing on the skill sets to manage complex treatment planning (multi‐modality planning, previous treatment assessment, differing fractionation regimens, motion management for SBRT, adaptive therapy, radiomics, etc.), repair gaps in quality and safety, and implement data driven, artificial intelligence. Given all of this, are radiation oncology physicists overpaid, as the CMS staff posited above? Will RO‐APM address that? While CMS staff may be right that the RO‐APM is a good thing for the US healthcare system, it will prove challenging for radiation oncology physicists.

The RO‐APM would test whether prospective episode‐based payments for radiation oncology episodes of care would reduce Medicare expenses while preserving or enhancing the quality of care for Medicare beneficiaries. This model is highly complex, and a review of it and AAPM’s thoughtful response are not within the scope of this editorial. However, there is one section at the heart of the proposed APM (page 34497 ‐ 98) that might offer an opportunity for a novel workforce and staffing research project to benefit clinical radiation oncology physicists:

“(page 34497) We identified 17 cancer types in Table 1 that meet our proposed criteria. These 17 cancer types are commonly treated with RT and Medicare claims data was sufficiently reliable to calculate prices for prospective episode payments that accurately reflect the average resource utilization for an episode. These cancer types are made up of specific ICD‐9 and ICD‐10 diagnosis codes…

We are proposing that the RO Model's included cancer types would include those that are commonly treated with RT and that can be accurately priced for prospective episode payments. An up‐to‐date list of cancer types would be kept on the RO Model website.

We propose to define the term “included cancer types” to mean the cancer types determined by the criteria set forth in Table 1, which are included in the RO Model test.”

We would maintain the list of ICD‐10 codes for included cancer types under the RO Model on the RO Model website.”

**Table 1:** Identified cancer types and corresponding icd‐9 and icd‐10 codes.

Cancer type	ICD‐9 Codes	ICD‐10 Codes
Anal Cancer	154.2X, 154.3x	C21.xx
Bladder Cancer	188xx	C67.xx
Bone Metastases	198.5x	C79.5x
Brain Metastases	198.3x	C79.3x
Breast Cancer	174.XX, 175.XX. 233.0x	C50.xx, D05.xx
Cervical Cancer	ISO.xx	C53.xx
CNS Tumors	191,xx, 192.0X, 192.1x, 192.2x, 192.3x. 192.8x, 192.9x	C70.xx, C71.xx, C72.xx
Colorectal Cancer	153.XX, 154.0x. 154. lx, 154.8x	C18.xx, C19.xx, C20.xx
Head and Neck Cancer	140.xx, 141.0x, Ml.lx, 141.2x, 141.3x, 141.4x, 141.5x, 141.6x, 141.8x, 141.9x, 142.0x, 142. lx, 142.2x, 142.8.x, 142.9x, 143.xx, 144.xx, 145.0x, 145.1x, 145.2x, 145.3x, 145.4x, 145.5x, 145.6.x, 145.8x, 145.9x, 146.0x, 146. lx, 146.2x, 146.3x, 146.4x, 146.5x, 146.6x, 146.7x, 146.8x, 146.9x 147.xx, 148.0x, 148.1x, 148.2.x, 148.3x, 148.8x, 148.9x, 149.XX, 160,0.x, 160. lx, 160.2x, 160.3x, 160.4x, 160.5x, 160.8x, 160.9x, 161.xx, 195.0x	COO.xx, COl.xx, C02.xx, C03.xx, C04.xx, C05.xx, C06.xx, C07.xx, C08.xx, C09.xx, ClO.xx, Cll.xx, C12.xx, C13.xx, C14.xx, C30.xx,C31.xx, C32.xx, C76.0x
Kidney Cancer	189.0x	C64.xx
Liver Cancer	155.XX, 156.0x, 156.1x, 156.2.x, 156.8x. 156.9x	C22.xx, C23.xx, C24.xx
Lung Cancer	162.0x. 162.2x, 162.3x. 162.4x, 162.5x, 162.8.x, 162.9.x. 165xx	C33.xx. C34.xx, C39.xx. C45..XX
Lymphoma	202.80,202.81, 202.82,202.83,202.84. 202.85, 202.86, 202.87,202.88, 203.80. 203.82,200.0x, 200.lx, 200.2x, 200.3x. 200.4x, 200.5x, 200.6x, 200.7x, 200.8x 201.xx, 202.0x, 202. lx, 202.2x, 202.4x. 202.7x, 273.3x	C81.xx, C82.xx, C83.xx, C84.xx. C85.xx, C86.xx, C88.xx, C91.4x
Pancreatic Cancer	157.xx	C25.xx
Prostate Cancer	185xx	C61.xx
Upper GI Cancer	150.XX, 151.XX, 152.XX	C15.xx, C16.xx, C17.xx
Uterine Cancer	179xx, 182.XX	C54.XX, C55.xx

The challenge is that each of these cancer types and ICD codes represent a wide range of disease presentations, and therefore, a wide range of costs to provide value‐based care. This wide range of costs exists for each cancer type within and among each provider type. We see three possible ways for the clinical physics community to respond, but, on retrospect, there are significant drawbacks:

- Perform a retrospective study reporting the CPT “physics” code service mix by institution for each cancer type. This would use the latest Abt survey to establish a knowledge base which could be cross‐linked to future manpower/staffing surveys. However, there is a good chance that tying physics value to identified cancer types will not work. If it were attempted, it might even have adverse consequences. And the effort would be expensive.
- Perform a prospective study measuring the preservice, intraservice, and postservice work performed by the clinical physicist to support each cancer type in a new type of survey with an entirely new design. Many larger institutions are already divided by service and cancer type, and the institutional data will need to be mined for work, effort and staffing. This type of study is extremely complex, and likely cost prohibitive as well.
- Unfortunately, in addition to cost and complexity, neither of the two studies above will carry much weight without support from our physician colleagues. We could easily waste time and resources if these studies are not readily accepted by both hospital administrators and our physician colleagues. It is vital that physicians have easy to use tools to persuade administrators that patient safety must come first and it can only be addressed by having adequate quality staff to treat patients. The interests of physicians and physicists are aligned on safety and quality of care. If the AAPM leadership could work with ASTRO and the endorsing organizations to update the “Safety is no Accident” staffing matrix, that would provide our physician colleagues with a very useful and simple tool. This pathway avoids associating workload and effort with CPT codes or cancer type, and assigns recommended staffing directly based on patient load categories. This study has the advantage of being less costly if designed and executed properly.

It is still possible that medical physics practice will revert to a situation similar to the early 1990s. The QMP credential might mean less if large numbers of non‐QMP physicists and physicist assistants were to perform much of the work now performed by QMPs. This could happen because there are a large number of non‐QMP physicists available for employment, and they, along with physicist assistants, would work for salaries equivalent to physicists in industry. This would further diminish the identity of the QMP and the ability of residency programs to attract quality candidates. Without fee‐for‐service reimbursement, medical physicists will find it much more difficult to defend their workload, staffing, and other needed resources to practice.

In all, CMS received 329 comments on the proposed RO‐APM and has proposed delaying its implementation. (https://www.regulations.gov/docket?D=CMS-2019-0101). However, there is a consensus in the community that the RO‐APM in some form is inevitable. AAPM leadership would be wise to be proactive and consider how to address this eventuality. The Abt studies were an order of magnitude in complexity above the ACR “Blue Book” recommendations. Another Abt study, even one of significantly greater complexity, may not prove as effective in the era of episodic payment models. Instead, a new approach based on collaboration with our physician colleagues, culminating in a revised SINA matrix, may be more relevant in the years ahead. We will need to develop a complementary strategy for our diagnostic imaging colleagues, in concert with the ACR.

